# Significance of tumour cell HLA-G5/-G6 isoform expression in discrimination for adenocarcinoma from squamous cell carcinoma in lung cancer patients

**DOI:** 10.1111/jcmm.12400

**Published:** 2015-02-16

**Authors:** Wei-Hua Yan, Di Liu, Hai-Yan Lu, Ying-Ying Li, Xia Zhang, Aifen Lin

**Affiliations:** a Medical Research Center, Taizhou Hospital of Zhejiang Province, Wenzhou Medical UniversityZhejiang, China; b Human Tissue Bank, Taizhou Hospital of Zhejiang Province, Wenzhou Medical UniversityZhejiang, China

**Keywords:** HLA-G, non-small-cell lung cancer, ROC

## Abstract

Human leucocyte antigen (HLA)-G has seven isoforms, of which HLA-G1-G4 are membrane-bound and HLA-G5-G7 are soluble. Previous studies reinforced HLA-G expression was strongly related to poor prognosis in different types of cancers. Among these studies, the monoclonal antibody (mAb) 4H84 was used which detects all HLA-G isoform heavy chain; unfortunately, leaves the specific types of isoforms expressed in lesions undistinguished and its clinical significance needs to be clarified. To explore clinical significance of lesion soluble HLA-G (sHLA-G) in non-small-cell lung cancer (NSCLC), mAb 5A6G7 recognizing HLA-G5/-G6 molecules was used. Tumour cell sHLA-G expression in 131 primary NSCLC lesions (66 squamous cell carcinoma, 55 adenocarcinoma and 10 adenosquamous carcinoma) were analysed with immunohistochemistry. Data showed that sHLA-G expression was observed in 34.0% (45/131) of the NSCLC lesions, which was unrelated to patient age, sex, lymph nodal status, tumour–node–metastasis stage and patient survival. However, tumour cell sHLA-G expression in lesions was predominately observed in adenocarcinoma lesions (73.0%, 40/55) which was significantly higher than that in squamous cell carcinoma (6.0%, 4/66) and adenosquamous carcinoma lesions (10.0%, 1/10, *P* < 0.001). The area under the receiver operating characteristic curve for lesion sHLA-G was 0.833 (95% CI: 0.754–0.912, *P* < 0.001) for adenocarcinoma *versus* squamous cell carcinoma. Our findings for the first time showed that tumour cell sHLA-G was predominately expressed in lung adenocarcinoma, which could be a useful biomarker to discriminate adenocarcinoma from squamous cell carcinoma in NSCLC patients.

## Introduction

Various strategies have been developed by tumour cells to avoid recognition and destruction by different immune effectors 1. One of the strategies used by tumour cells to elude host immune response is the alterations in the expression and/or function of the major histocompatibility complex class I antigens, such as induction of the expression of the non-classical human leucocyte antigen (HLA) class I antigen HLA-G 2,3.

Unlike classical HLA class I antigens, HLA-G primary transcript could generate seven isoforms by alternative splicing. Among these isoforms, HLA-G1, -G2, -G3 and -G4 are membrane-bound, while HLA-G5, -G6, and -G7 are soluble 4. HLA-G expression was firstly observed in cytotrophoblasts and its tissue distribution was very limited in physiological conditions; however, HLA-G can be neoexpressed in pathological conditions such as cancers 5,6. In the scenario of malignancies, ectopic induction of HLA-G expression has been observed in various types of tumours including haematological and solid tumours, where different sources of HLA-G expression exist such as on the cell surface, secreted or incorporated into tumour-derived exosomes 7–9. HLA-G expression was more frequently observed in tumour lesions with advanced stage, and a poor prognosis 10.

In non-small-cell lung cancer (NSCLC), our previous study showed that lesion HLA-G expression was found to be significantly associated with stage of the disease 11. Yie *et al*. 12 addressed that NSCLC primary lesion HLA-G expression was significantly associated with the poor prognosis and shorter survival time. Similar findings was also observed in other malignancies such as gastric carcinomas, colorectal cancer and oesophageal squamous cell carcinoma patients that lesion HLA-G expression was strongly correlated with disease stage and poor prognosis and that HLA-G could be an independent prognostic factor 13–15. In all previous studies, the HLA-G expression in malignant lesions were mostly detected by mAb 4H84 such as in the study of NSCLC 11, and another mAb HGY which was developed and applied to probe HLA-G by Yie's laboratory 12. mAb 4H84, an IgG1 HLA-G-specific antibody which could detect denatured HLA-G heavy chain of all HLA-G isoforms including HLA-G1-HLA-G7 16. Given their a broad specificity for HLA-G isoform detection, results obtained by using mAb 4H84 and HGY could only read the combination of certain types of HLA-G isoform expression; however, the fine types of isoforms expressed in tumour lesions remains undistinguished.

To make a fine exploration of lesion sHLA-G expression in NSCLC patients, in this study, the 5A6G7, an IgG1 mAb which probes denatured heavy chain of HLA-G5/HLA-G6 (sHLA-G) molecules, was used 16. Lesion sHLA-G expression in 131 primary NSCLC lesions and corresponding adjacent normal tissues were analysed with immunohistochemistry. Our findings showed that sHLA-G expression was predominately in lung adenocarcinoma which could be a useful biomarker to discriminate lung adenocarcinoma from adenosquamous carcinoma patients.

## Materials and methods

### Tissue samples

One hundred and sixty-nine samples including 131 primary NSCLC lesions and 38 case-matched adjacent normal lung tissues were consecutively collected from patients with lung cancer diagnosed and treated at Taizhou Hospital of Zhejiang Province, Wenzhou Medical University between Nov 22, 2004, and July 8, 2010. Among 131 NSCLC patients, there were 66 squamous cell carcinoma, 55 adenocarcinoma and 10 adenosquamous carcinoma patients. The clinicopathological findings were documented according to the classification of malignant tumours by World Health Organization and International Union Against Cancer Tumor-Node-Metastasis (TNM) staging system 17. None of the patients received radiotherapy, chemotherapy or other medical interventions before the study.

Of 131 cases, 39 (29.8%) were with stage I, 72 (55.0%) were with stage II, 15 (11.5%) were with stage III and 5 (3.8%) were with stage IV respectively. Among these cases, 123 patients were available for a follow-up study. Patients in stages I-III received surgical resection followed by either a radiotherapy and/or chemotherapy, while patients in stages IV received a systemic chemotherapy and/or radiotherapy. The follow-up period was 8 years or until death. The average follow-up for all patients was 38 months (range, 4–96 months), and during the entire period, there were 72 (55.0%) cancer-related deaths including 13 (33.3%), 41 (56.9%), 13 (86.7%) and 5 (100%) patients with stage I, II, III and IV respectively.

Patient data including age, gender, date of initial diagnosis, lymph nodal status and TMN stage and survival time were documented. All samples were obtained from the primary lesions of the lung cancer. No specimens from metastatic disease sites were included in the study. All tissue specimens underwent a microscopic confirmation for pathological features prior to their inclusion in the study. This study was performed following an Institutional Ethics Review Board approved protocol to investigate molecular markers relevant to lung cancer pathogenesis, and informed consent was obtained from all patients.

### Immunohistochemistry and staining evaluation

Immunohistochemistry was performed with a standard method as previously reported 11. The anti-HLA-G5/-G6 (sHLA-G) antibody 5A6G7 (1:300; Exbio, Prague, Czech Republic) was applied to detect the sHLA-G expression in 131 NSCLC cases, and mAb 4H84, an IgG1 mAb detecting denatured HLA-G heavy chain of all HLA-G isoforms (1:300; Exbio) was used to detect total HLA-G expression in 39 case-matched samples including 19 cases of squamous cell carcinoma, 17 cases of adenocarcinoma and 3 cases of adenosquamous carcinoma.

A sample was scored as positive when the percentage of stained lung cancer cells in the entire lesion was >5% and negative when the percentage was ≤5%. For the evaluation of sHLA-G expression, two independent observers (Lin A and Zhang X) assessed sHLA-G positivity semiquantitatively, without previous knowledge of clinicopathological data. The percentage of positive cells was assigned a value based on the presence or absence of sHLA-G staining, irrespective of staining intensity.

### Flow cytometric analysis

Fifteen NSCLC patients were subjected to the peripheral monocyte intracellular sHLA-G expression analysis. PBMC were gated with PE-labelled anti-CD45 (BD, San Jose, CA, USA) and intracellular sHLA-G expression on monocytes was analysed. The mAb FITC-2A12, an IgG1 mAb detecting intracellular HLA-G5/-G6 molecules (Exbio) was used. Intracellular analysis of sHLA-G was performed after fixation and permeabilization according to the manufacturer's recommendation (eBioscience, San Diego, CA, USA). Flow cytometry was performed on a FACSCalibur and analysed using the CellQuest (BD Biosciences, San Jose, CA, USA) software packages.

### Tissue protein extraction and Western blot analysis

Tissue protein extraction and Western blot was performed with a standard method as previously reported 11. The anti-HLA-G5/-G6 (sHLA-G) antibody 5A6G7 (1:1000; Exbio) was applied to detect the NSCLC lesion sHLA-G expression in 10 case-matched samples including five cases of squamous cell carcinoma, and five cases of adenocarcinoma. Anti-Calnexin mAb (1:1000, a house keeping protein, molecular weight 90 kD, Stressgen, Glanford Ave, Victoria, BC, Canada) was used as an internal control.

### sHLA-G ELISA

Plasma sHLA-G concentrations were determined in 30 case-matched NSCLC patients with the sHLA-G-specific ELISA kit (sHLA-G kit; Exbio). Each sample (50 μl) was measured in triplicates. The optical densities were measured at 450 nm (Spectra Max 250; Molecular Devices, Sunnyvale, CA, USA). The final concentration was determined by optical density according to the standard curves (range: 0–125 U/ml). When the concentration exceeds 125 U/ml, diluted samples were used and dilution factors were considered to calculate the sHLA-G concentration. The detection limits were 1 U/ml. Details of the performance were according to the manufacture's instruction.

### Statistical analysis

Statistical analysis was performed with SPSS 13.0 software (SPSS, Inc., Chicago, IL, USA). Correlations between the lung cancer lesion sHLA-G expression and clinical parameters were calculated with Pearson chi-squared test. Overall patient survival was evaluated from the date of diagnosis to the date of last follow-up (censored) or date of patient death (event). Survival probabilities were calculated using the Kaplan–Meier method. Differences between survival curves were analysed by the log-rank test. Correlation between survival time and multiple clinicopathological variables by univariate and multivariate analyses were conducted using the Cox regression analysis. The feasibility of using tumour cell sHLA-G expression as a potential biomarker for distinguish patients with squamous cell carcinoma from adenocarcinoma was assessed using the receiver operating characteristic (ROC) curve analysis. The areas under the ROC curve were calculated and subjected to statistical analysis. *P* < 0.05 was considered to be significant.

## Results

### Tumour cell sHLA-G expression and its relation to clinical parameters in primary NSCLC lesions

Among 131 NSCLC patients, there were 66 squamous cell carcinoma, 55 adenocarcinoma and 10 adenosquamous carcinoma patients. Lesion sHLA-G expression in these NSCLC patients was analysed with immunohistochemistry using mAb 5A6G7.

**Fig 1:**
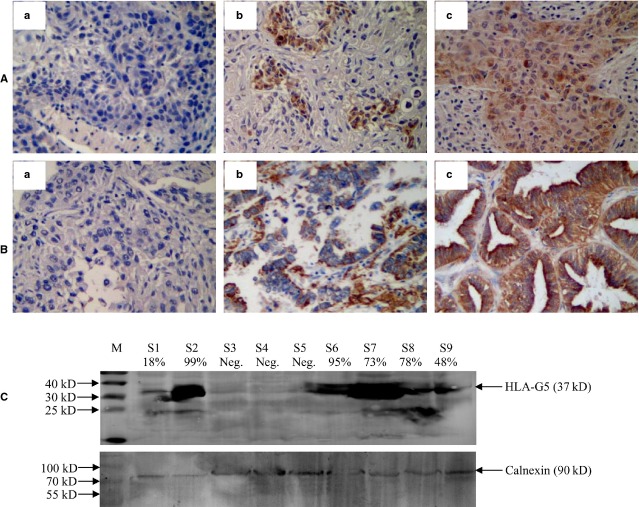
Immunohistochemical staining of tumour cells sHLA-G expression in NSCLC lesions. (A) negative (a) and positive (b, c) expression of sHLA-G in lung squamous cell carcinoma lesions; (B) negative (a) and positive (b, c) expression of sHLA-G in lung adenocarcinoma lesions; HLA-G mAb 5A6G7 (1:300) was used to detect the lesion sHLA-G expression. sHLA-G expression was considered as negative when the stained cell percentage was ≤ 5%. Original magnification: 100 × . (C), Western blot analysis of HLA-G expression. The analysis was performed with the sHLA-G mAb 5A6G7 (1:1000). M, molecular weight ladder; samples of patient (S3, 4, 5) were from HLA-G negative, and patient (S1, 6, 7, 8, 9) was from sHLA-G positive NSCLC patients. The degree of HLA-G expression was shown in brackets according to the case-matched immunohistochemistry. The house keeping protein Calnexin was used as an internal control (molecular weight: 90 kD).

In the malignant tumour sections, the intensity of staining for tumour cells was varied from tumour to tumour and from one area to another within the same tumour. Heterogeneous staining for tumour cells was noted in all histological types of NSCLC lesions (Fig.1A and B). Furthermore, HLA-G expression in 10 samples was tested by Western blot. Our data showed that, immunohistochemistry results were highly consistent with that of the Western blot analysis with mAb 5A6G7 (Fig.1C).

To test the consistency of the reactivity of mAb 4H84 (detecting denatured HLA-G heavy chain of all HLA-G isoforms) and mAb 5A6G7 (detecting denatured HLA-G5/-G6 heavy chain), 39 cases-matched NSCLC lesions including 13 positive and 26 negative samples detected with mAb 5A6G7, were performed with immunohistochemistry analysis. Among these samples, 14 and 10 samples were negative and positive with both mAb 4H84 and mAb 5A6G7 respectively. Twelve samples were positive with mAb 4H84 but not mAb 5A6G7, and three of them were positive with mAb 5A6G7 but not with mAb 4H84 (Table S1).

Overall, 34.0% (45/131) NSCLC tumour lesions were classified as sHLA-G positive. Interestingly, tumour cell sHLA-G expression was found predominately in adenocarcinoma patients (73.0%, 40/55) which was significantly higher than that in squamous cell carcinoma (6.0%, 4/66) and in adenosquamous carcinoma patients (10.0%, 1/10, *P* < 0.001). Furthermore, sHLA-G expression was more frequently observed in female patients (77.0%, 20/26) than that in male patients (24.0%, 25/105, *P* < 0.001; Table1).

**Table 1:** Association of tumour cell sHLA-G expression in the whole cohort of primary NSCLC lesions with clinicopathological parameters

Variables	No. of cases	sHLA-G expression		P*
		Negative (%)	Positive (%)	
Histological type	131	86 (66.0)	45 (34.0)	
Squamous cell carcinoma	66	62 (94.0)	4 (6.0)	<0.01
Adenocarcinoma	55	15 (27.0)	40 (73.0)	
Adenosquamous carcinoma	10	9 (90.0)	1 (10.0)	
Gender				
Male	105	80 (76.0)	25 (24.0)	<0.01
Female	26	6 (23.0)	20 (77.0)	
Age				
≤median (60 years)	71	48 (68.0)	23 (32.0)	0.608
>median	60	38 (63.0)	22 (37.0)	
Nodal status				
Negative	68	46 (68.0)	22 (32.0)	0.617
Positive	63	40 (63.0)	23 (37.0)	
TNM stage				
I	39	25 (64.0)	14 (36.0)	0.537
II	72	50 (69.0)	22 (31.0)	
III	15	9 (60.0)	6 (40.0)	
IV	5	2 (40.0)	3 (60.0)	

* Comparison of sHLA-G expression status between or among each variable using the Pearson chi-squared test.

tumour cell sHLA-G expression in NSCLC lesions was unrelated to patient age, lymph nodal status, TNM stage either in all NSCLC patients or in patients with squamous cell carcinoma and adenocarcinoma (Table1, Tables S2 and S3). For the limited size, where only 10 cases of the adenosquamous carcinoma were in the cohort, the statistical analysis for adenosquamous carcinoma in this study was not performed.

Thirty plasma samples obtained from case-matched NSCLC patients before operation were analysed for concentration of sHLA-G expression. The median sHLA-G level in these patients was 23.6 U/ml (range: 6.60–220.9 U/ml), which was not correlated with the case-matched lesion sHLA-G expression (*P* = 0.276, data not shown). To investigate the possible source of plasma sHLA-G expression, 15 peripheral blood samples from another cohort of NSCLC patients were obtained for flow cytometry analysis. Results indicated that plasma sHLA-G might be released from peripheral blood monocytes as the flow cytometry data showed intracellular sHLA-G was expressed in 82.2 ± 7.8% monocytes (range: 70.2–96.5%, Fig. S1).

### Tumour cell sHLA-G expression and clinical parameters relating to survival in NSCLC patients

To investigate the relationship between clinical parameters and the clinical outcome of NSCLC patients, patient survival relating to sHLA-G expression, patient sex and age, lymph nodal status and TNM stage was analysed.

In whole cohort, the mean survival time for the sHLA-G-negative NSCLC patients (total *N* = 82, event *N* = 47) was 57.4 months (95% CI: 48.9–66.0 months), and for the sHLA-G-positive NSCLC patients (total *N* = 41, event *N* = 26) was 53.2 months (95% CI: 41.3–65.2 months). Though the mean survival time for lesion sHLA-G-positive patients was less than that of sHLA-G-negative patients, no significance was observed between the two groups (*P* = 0.559, Fig. S2A). Moreover, lesion sHLA-G expression status was not related to the survival of the patients either with squamous cell carcinoma (*P* = 0.151; Fig. S2B) or adenocarcinoma (*P* = 0.919; Fig. S2C).

Among other factors such as histological classification of lung cancer, patient age, sex, lymph nodal status and TNM stage, both lymph nodal status and TNM stage was significantly associated with NSCLC patient survival.

For the histological classification of lung cancer, though not reach a statistic significance, the mean survival time for the squamous cell carcinoma patients was 59.3 months (total *N* = 62, event *N* = 34; 95% CI: 50.2–68.4 months), for the adenocarcinoma patients was 53.8 months (total *N* = 51, event *N* = 31; 95% CI: 43.0–64.6 months), and for the adenosquamous carcinoma patients was 31.8 months (total *N* = 10, event *N* = 7; 95% CI: 14.6–49.0 months; *P* = 0.126; Fig. S3A). The mean survival time for the lymph nodal negative patients was 67.7 months (total *N* = 63, event *N* = 28; 95% CI: 58.1–77.4 months), for the nodal positive patients was 43.2 months (total *N* = 58, event *N* = 42; 95% CI: 34.4–52.0 months; *P* < 0.001; Fig. S3B). The mean survival time for the patients with TNM stage I was 72.5 months (total *N* = 37, event *N* = 13; 95% CI: 59.4–85.6 months), for the patients with TNM stage II was 55.0 months (total *N* = 67, event *N* = 41; 95% CI: 46.4–63.6 months), for the patients with TNM stage III was 25.4 months (total *N* = 14, event *N* = 13; 95% CI: 14.2–36.5 months), and for the patients with TNM stage IV was 28.2 months (total *N* = 5, event *N* = 5; 95% CI: 8.0–48.4 months; *P* < 0.001; Fig. S3C).

Furthermore, on univariate analysis, factors including nodal status and TNM stage showed a significantly higher hazard ratio for a poor prognosis in the whole cohort of the NSCLC patients. Moreover, multivariate analysis revealed that, both nodal status (*P* = 0.034) and TNM stage (*P* < 0.001) were independent prognostic factors for NSCLC patients. However, only in squamous cell carcinoma patients, the TNM stage remains an independent prognostic factor as multivariate analysis result indicated (Table S4).

### ROC analysis for tumour cell sHLA-G as a biomarker for patients with squamous cell carcinoma and adenocarcinoma

**Fig 2:**
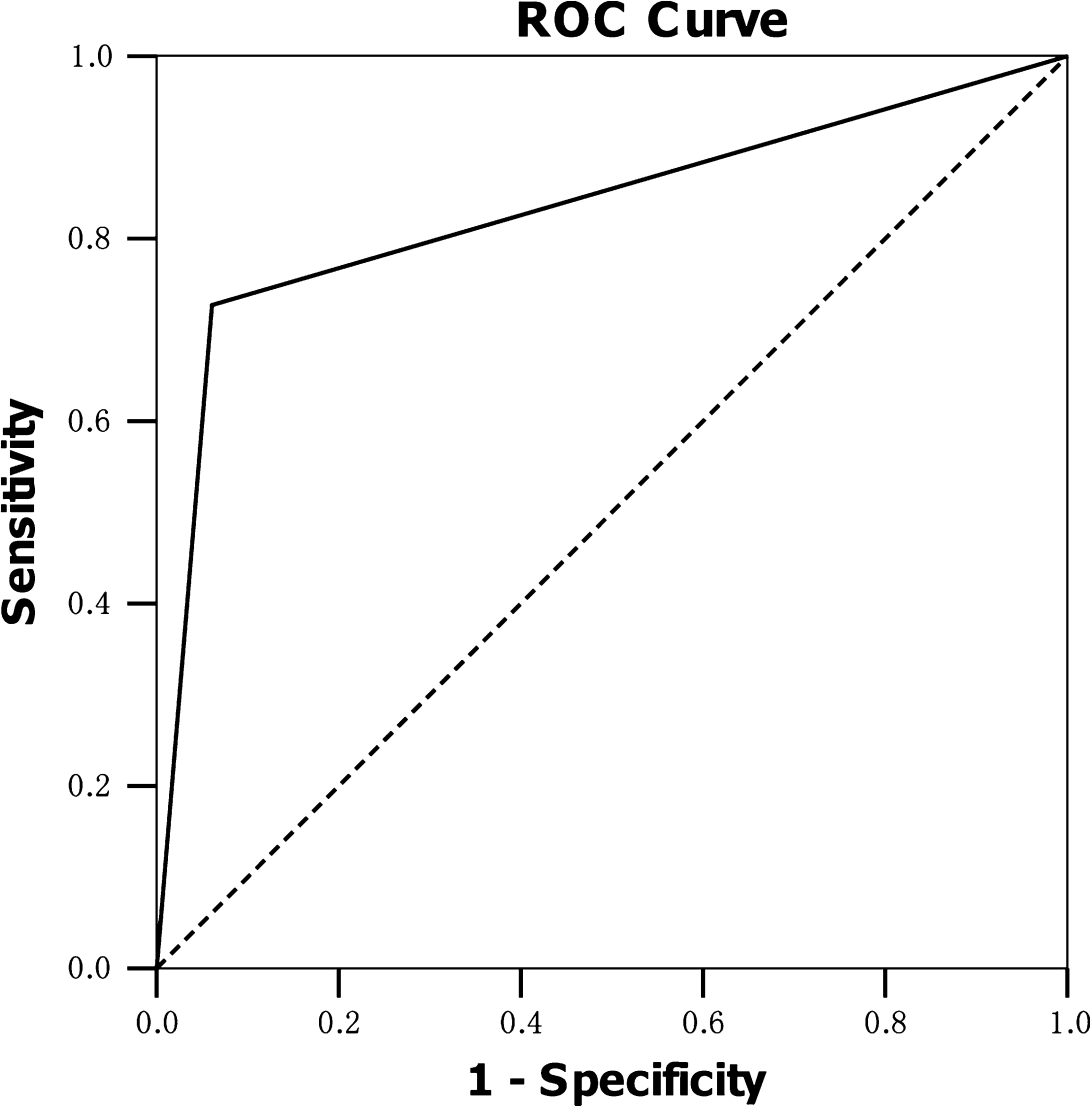
Receiver operating characteristic (ROC) curve analysis of lesion sHLA-G expression for discriminating adenocarcinoma (*n* = 55) from squamous cell carcinoma (*n* = 66). Area under the curve (AUC) = 0.833, (95% CI 0.754–0.912, *P* < 0.001).

Receiver operating characteristic curves were used to evaluate the performance of sHLA-G in discriminate patients with squamous cell carcinoma and adenocarcinoma. The area under ROC curve for tumour cell sHLA-G expression was 0.833 (95% CI, 0.754-0.912, *P* < 0.001) for patient with adenocarcinoma *versus* squamous cell carcinoma (Fig.2).

## Discussion

Ectopic induction of HLA-G expression has been observed in various malignant tissues and cells and roles of aberrant HLA-G expression in malignancies have been extensively investigated 18. HLA-G could directly inhibit immune cell cytolysis, induce apoptosis and generation of regulatory cells through receptor binding and/or trogocytosis 10. Moreover, HLA-G expression was frequently observed in tumour lesions with advanced stage, and was associated with a higher invasive or metastatic potential for tumour cells, or an unfavourable prognosis in tumour patients, indicating that HLA-G expression is of multiple effects during the progression of malignancies 6.

Human leucocyte antigen-G can generate seven alternative mRNAs by alternative splicing and encode four membrane-bound (HLA-G1–HLA-G4) and three soluble isoforms (HLA-G5–HLA-G7) 19. A high frequency of malignant lesion HLA-G expression and increased peripheral blood sHLA-G levels have been detected in haematological and solid tumours 8,9. Among studies for solid tumours, lesion HLA-G expression detected by immunohistochemistry was widely performed, where HLA-G is frequently observed in cancers such as ovarian, breast, endometrial and hepatocellular carcinoma, *etc*. 20–23. In all these studies, the HLA-G expression in malignant lesions were mostly detected by mAb 4H84, and another mAb HGY which was developed and applied to probe HLA-G by Yie's laboratory 12. mAb 4H84, an HLA-G-specific antibody detect denatured HLA-G heavy chain of all HLA-G isoforms including HLA-G1-HLA-G7, and HGY could also be specific for HLA-G while the specificity for HLA-G isoforms remains unclear 4,16. As a result, HLA-G detected with mAb 4H84 and HGY could only present the combination of certain types of HLA-G isoform (total HLA-G) expression; however, leaves the type of isoform expression in tumour lesions undistinguished, such as the status of lesion sHLA-G isoform expression.

As a result of the mAb 5A6G7 could not discriminate the isoforms between HLA-G5 and HLA-G6 in immunohistochemistry analysis, a Western blot was used to investigate the pattern of HLA-G5 and HLA-G6 expression in lesions in this study, according to their molecular weight with 37 kD and 27 kD respectively 24. We found that HLA-G5 is the main isoform of lesion sHLA-G expression, and this is strongly agreed to the results of immunohistochemistry as shown in Figure1C.

We further explored the reactivity consistency between mAb4H84 (detecting denatured HLA-G heavy chain of all HLA-G isoforms) and mAb 5A6G7 (detecting denatured HLA-G heavy chain of HLA-G5/-G6 isoforms) in 39 case-matched NSCLC lesions with immunohistochemistry. Among 26 samples negative with mAb 5A6G7, 14 of them were negative with both mAb4H84 and mAb 5A6G7, and 12 of them were positive with both mAb4H84, indicating that HLA-G isoforms other than HLA-G5/-G6 might be present in these 12 NSCLC lesions. Among 13 samples positive with mAb 5A6G7, 10 of them were also positive with both mAb4H84, except for three lesion which were positive with mAb 5A6G7 but not with mAb4H84. The inconsistency between the reactivity between mAb 5A6G7 and mAb4H84 in these three samples might be result from too weak expression of sHLA-G to be detected by mAb4H84.

In this study, our data revealed that lesion tumour cell sHLA-G expression detected with mAb 5A6G7 was predominately observed in patients with adenocarcinoma, which was dramatically higher than that in squamous cell carcinoma and in adenosquamous carcinoma patients. Our findings showed that lesion sHLA-G expression was not associated with clinical parameters such as TNM stage, lymph nodal status and patient survival. As to the lesion total HLA-G expression (with mAb 4H84), which was observed with no difference between patient with adenocarcinoma and squamous cell carcinoma, indicating that total HLA-G expression did not vary dramatically in these two histological type NSCLC patients; however, lesion total HLA-G expression was found to be significantly associated with disease clinical TNM stage and significantly associated with the poor prognosis and shorter survival time 11,12. Given the importance of HLA-G expression in malignancies, the different tissue distribution and clinical significance between lesion total HLA-G and sHLA-G expression makes it necessary to further study the fine exploration of specific HLA-G isoform expression and then its clinical implications.

More importantly, in this study, ROC analysis showed that lesion tumour cell sHLA-G expression is of strong power to discriminate patients with adenocarcinoma from squamous cell carcinoma. The area under ROC curve for lesion sHLA-G expression was 0.833 (*P* < 0.001). In this context, a previous study reported that lesion total HLA-G (with mAb 4H84) expression in endometrial carcinomas to distinguish metastatic from non-metastatic endometrial carcinoma is powerful as the area under ROC curve was 0.75 23. These data indicated that lesion total HLA-G or specific HLA-G isoform expression may serve as a clinical marker for tumour metastasis or hiso-type discrimination.

Besides lesion sHLA-G, the significance of peripheral blood sHLA-G in many types of malignancies was investigated 6. Our previous data showed that plasma sHLA-G levels were significantly associated with the NSCLC disease stage, where sHLA-G in stage IV was much higher than that in stage I or in stage II 11. Schütt *et al*. 25 reported that plasma sHLA-G was exclusively elevated in NSCLC, especially in patients with squamous cell carcinoma. Patients with sHLA-G <40 ng/ml showed prolonged overall survival. However, no such significance was observed in this study for the lesion sHLA-G expression either in the whole cohort or in adenocarcinoma, squamous cell carcinoma, adenosquamous carcinoma respectively. The reasons that, as reported before, there was no correlation between the lesion HLA-G expression and plasma sHLA-G level in the same patient, which was reinforced by a study on melanoma that sHLA-G molecules were preferentially released by peripheral blood rather than melanoma cells, and this was supported by the fact that peripheral blood monocytes are the predominant source of HLA-G5 production 11,26,27. In this context, our findings supported the fact that plasma sHLA-G might be released from peripheral blood monocytes as the flow cytometry data showed intracellular sHLA-G was expressed in 82.2 ± 7.8% monocytes (Fig. S1). Multiple factors influenced sHLA-G expression was observed, such as polymorphisms both in the promoter and in the 3′ untranslated region that modify the affinity of gene targeted sequences for transcriptional or post-transcriptional factors, epigenetic modification, cytokines, growth factors and hormones 28,29. Given the fact that plasma sHLA-G levels varies with a wide range among different individuals, and no association between tumour lesion sHLA-G expression and plasma sHLA-G levels both in malignant patients 11,27 and in a mouse model 30, more studies are needed to elucidate the clinical significance of both tumour lesion and peripheral blood sHLA-G expression and precise mechanisms involved in the regulation of HLA-G expression.

Taken together, our findings revealed that lesion tumour cell sHLA-G is predominately expressed in lung adenocarcinoma which could be a fair discriminator for adenocarcinoma *versus* squamous cell carcinoma. Unlike lesion total HLA-G expression which is strongly correlated with NSCLC clinical stage and poor prognosis, further studies are warranted to unravel the potential biological function of sHLA-G expression in malignancies.

## Supporting Information

**Figure S1** Flow cytometry analysis of intracellular sHLA-G expression in NSCLC patient peripheral monocytes with mAb 2A12.

**Figure S2** Kaplan–Meier survival analysis for tumour cell sHLA-G expression in NSCLC patients.

**Figure S3** Kaplan–Meier survival analysis for NSCLC patients.

**Table S1** Pattern of reactivity of mAb 4H84 and 5A6G7 in case-match NSCLC lesions.

**Table S2** Association of tumour cell sHLA-G expression in squamous cell carcinoma lesions with clinicopathological parameters.

**Table S3** Association of tumour cell sHLA-G expression in adenocarcinoma lesions with clinicopathological parameters.

**Table S4** Multivariate analysis of prognostic factors for overall survival in lung cancer patients.
