# Supplementary figures and images for: Significance of tumour cell HLA-G5/-G6 isoform expression in discrimination for adenocarcinoma from squamous cell carcinoma in lung cancer patients
Source: J Cell Mol Med. 2015 Feb 16;19(4):778–85. doi: 10.1111/jcmm.12400 (PMC4395192; doi:10.1111/jcmm.12400)

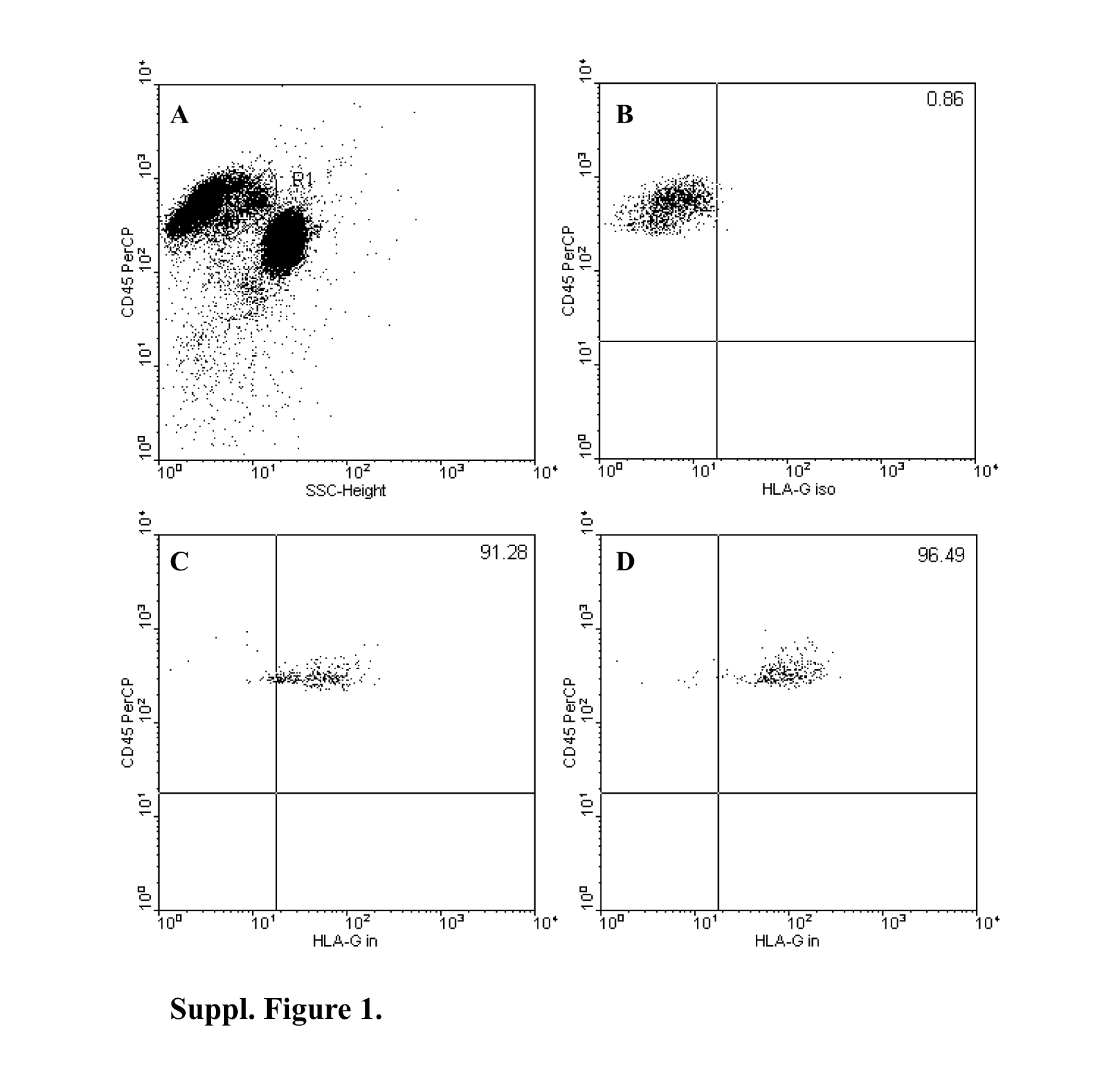

Supplement: Supplementary file 1 [file jcmm0019-0778-sd1.tif]

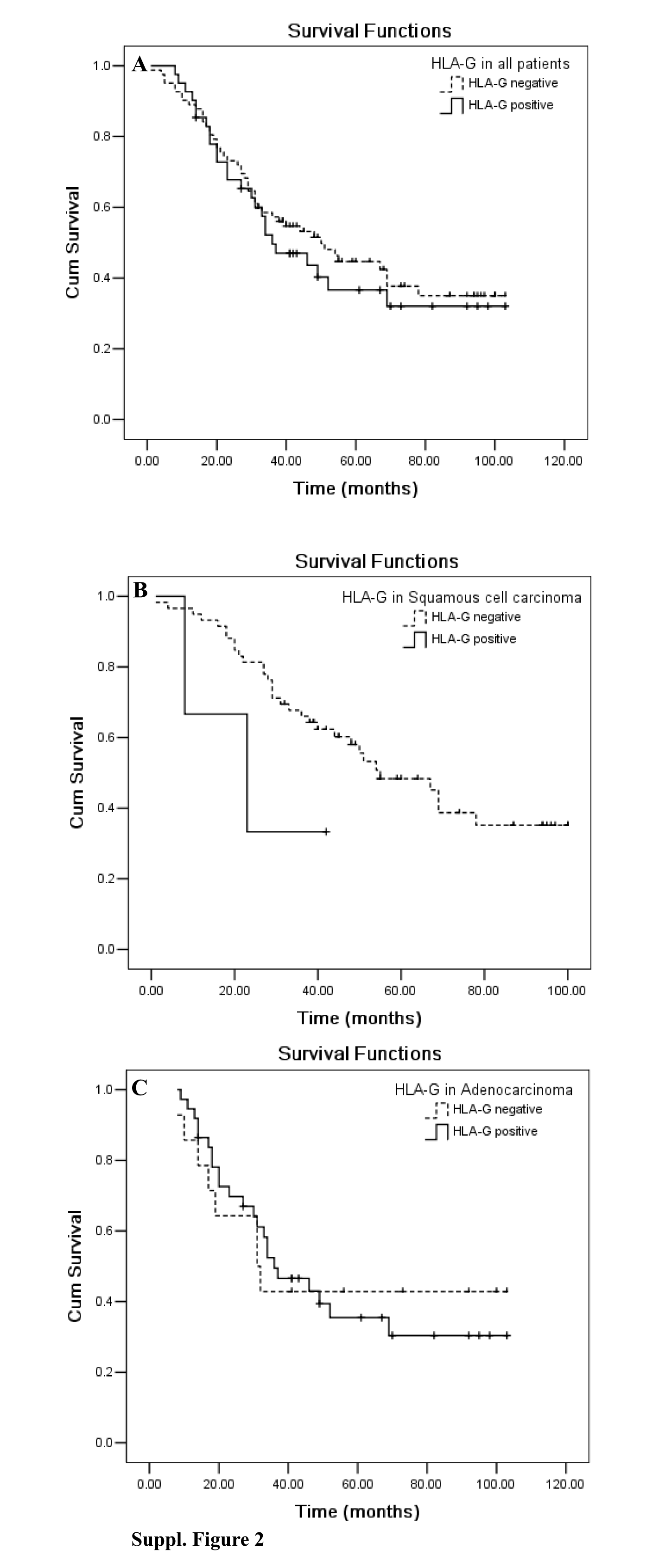

Supplement: Supplementary file 2 [file jcmm0019-0778-sd2.tif]

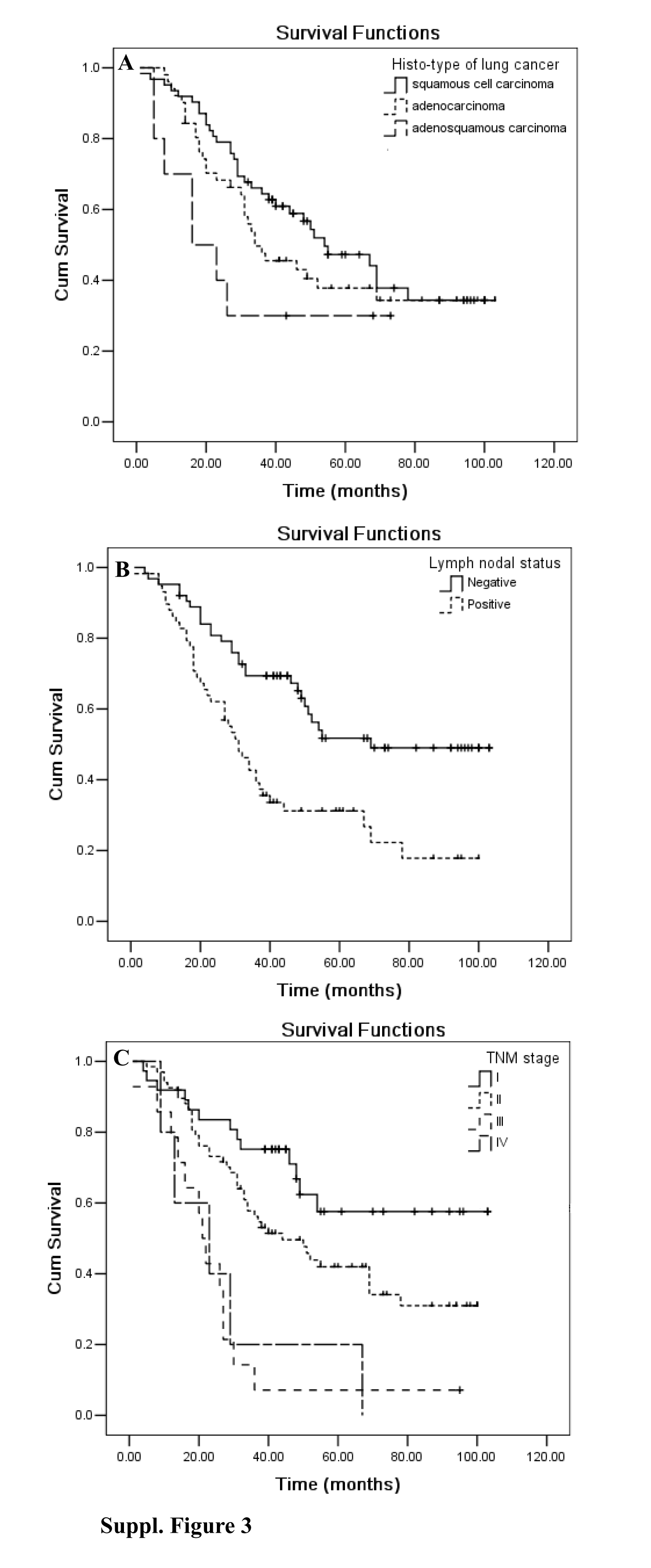

Supplement: Supplementary file 3 [file jcmm0019-0778-sd3.tif]
