# Supplementary material for: Significance of tumour cell HLA-G5/-G6 isoform expression in discrimination for adenocarcinoma from squamous cell carcinoma in lung cancer patients
Source: J Cell Mol Med. 2015 Feb 16;19(4):778–85. doi: 10.1111/jcmm.12400 (PMC4395192; doi:10.1111/jcmm.12400)
Supplement: Supplementary file 4 [file jcmm0019-0778-sd4.doc]

| Suppl. Table 1. Pattern of reactivity of mAb 4H84 and 5A6G7 in case-match NSCLC lesions | | |
| --- | --- | --- |
| mAb 4H84 | mAb 5A6G7 | Cases |
| **-** | **-** | 14 |
| **+** | **+** | 10 |
| **+** | **-** | 12 |
| **-** | **+** | 3 |
