# Supplementary material for: Significance of tumour cell HLA-G5/-G6 isoform expression in discrimination for adenocarcinoma from squamous cell carcinoma in lung cancer patients
Source: J Cell Mol Med. 2015 Feb 16;19(4):778–85. doi: 10.1111/jcmm.12400 (PMC4395192; doi:10.1111/jcmm.12400)
Supplement: Supplementary file 5 [file jcmm0019-0778-sd5.doc]

| Suppl. Table 2. Association of tumor cell sHLA-G expression in squamous cell carcinoma lesions with clinicopathological parameters | | | | |
| --- | --- | --- | --- | --- |
| Variables | No. of cases | sHLA-G expression | |  |
|  |  | Negative (%) | Positive (%) | *p** |
| Squamous cell carcinoma | 66 | 62 (94.0) | 4 (6.0) |  |
| Gender |  |  |  |  |
| Male | 64 | 61 (95.3) | 3 (4.7) | 0.008 |
| Female | 2 | 1 (50.0) | 1 (50.0) |  |
| Age |  |  |  |  |
| ≤median (60 years) | 38 | 36 (94.7) | 2 (5.3) | 0.752 |
| >median | 28 | 26 (82.9) | 2 (7.1) |  |
| Nodal status |  |  |  |  |
| Negative | 33 | 32 (97.0) | 1 (3.0) | 0.302 |
| Positive | 33 | 30 (91.0) | 3 (9.0) |  |
| TNM stage |  |  |  |  |
| I | 16 | 16 (100.0) | 0 (0.0) | 0.487 |
| II | 42 | 38 (90.5) | 4 (9.5) |  |
| III | 6 | 6 (100.0) | 0 (0.0) |  |
| IV | 2 | 2 (100.0) | 0 (0.0) |  |
| **^*^**Comparison of sHLA-G expression status between or among each variable using the Pearson chi-square test. | | | | |
