# Supplementary material for: Significance of tumour cell HLA-G5/-G6 isoform expression in discrimination for adenocarcinoma from squamous cell carcinoma in lung cancer patients
Source: J Cell Mol Med. 2015 Feb 16;19(4):778–85. doi: 10.1111/jcmm.12400 (PMC4395192; doi:10.1111/jcmm.12400)
Supplement: Supplementary file 6 [file jcmm0019-0778-sd6.doc]

| Suppl. Table 3．Association of tumor cell sHLA-G expression in adenocarcinoma lesions with clinicopathological parameters | | | | |
| --- | --- | --- | --- | --- |
| Variables | No. of cases | sHLA-G expression | |  |
|  |  | Negative (%) | Positive (%) | *p** |
| Adenocarcinoma | 55 | 15 (27.3) | 40 (72.7) |  |
| Gender |  |  |  |  |
| Male | 33 | 11 (33.3) | 22 (66.7) | 0.216 |
| Female | 22 | 4 (18.2) | 18 (81.8) |  |
| Age |  |  |  |  |
| ≤median (60 years) | 27 | 6 (22.2) | 21 (77.8) | 0.860 |
| >median | 28 | 9 (32.1) | 19 (67.9) |  |
| Nodal status |  |  |  |  |
| Negative | 29 | 9 (31.0) | 20 (69.0) | 0.508 |
| Positive | 26 | 6 (23.1) | 20 (76.9 ) |  |
| TNM stage |  |  |  |  |
| I | 19 | 6 (31.8) | 13 (68.4) | 0.256 |
| II | 27 | 9 (33.3) | 18 (66.7) |  |
| III | 6 | 0 (0.0) | 6 (100.0) |  |
| IV | 3 | 0 (0.0) | 3 (100.0) |  |
| **^*^**Comparison of sHLA-G expression status between or among each variable using the Pearson chi-square test. | | | | |
