# Supplementary material for: Significance of tumour cell HLA-G5/-G6 isoform expression in discrimination for adenocarcinoma from squamous cell carcinoma in lung cancer patients
Source: J Cell Mol Med. 2015 Feb 16;19(4):778–85. doi: 10.1111/jcmm.12400 (PMC4395192; doi:10.1111/jcmm.12400)
Supplement: Supplementary file 7 [file jcmm0019-0778-sd7.doc]

| Suppl. Table 4． Multivariate analysis of prognostic factors for overall survival in lung cancer patients | | | | | | | |
| --- | --- | --- | --- | --- | --- | --- | --- |
| Variables | Categories |  | Univariate Analysis | |  | Multivariate Analysis | |
|  |  |  | Overall survival | |  | Overall survival | |
|  |  |  | HR (95% CI) | *P** |  | HR (95% CI) | *P** |
| Histo-type | Adeno *vs* Squamous |  | 1.28 (0.79 – 2.08) | 0.321 |  |  |  |
|  | Adenosquamous *vs* Adeno |  | 1.73 (0.76 – 3.94) | 0.192 |  |  |  |
|  | Adenosquamous *vs* Squamous |  | 1.49 (0.99 – 2.24) | 0.059 |  |  |  |
|  |  |  |  |  |  |  |  |
| Total group | Sex (male *vs* female) |  | 0.99 (0.56 – 1.79) | 0.989 |  |  |  |
|  | Age (>60 years *vs* ≤60 years) |  | 0.70 (0.43 – 1.11) | 0.129 |  | 0.83 (0.51 – 1.36) | 0.463 |
|  | Nodal status (pN+ *vs* pN–) |  | 2.31 (1.43 – 3.75) | 0.001 |  | 1.76 (1.05 – 2.97) | 0.034 |
|  | TNM stage (III/IV *vs* I/II) |  | 3.60 (2.08 – 6.24) | 0.000 |  | 2.92 (1.64 – 5.21) | 0.000 |
|  | sHLA-G (Pos. *vs* neg.) |  | 1.15 (0.71 – 1.88) | 0.563 |  |  |  |
|  |  |  |  |  |  |  |  |
| Squamous cell carcinoma | Sex (male *vs* female) |  | 0.05 (0.00 – 642) | 0.529 |  |  |  |
|  | Age (>59 years *vs* ≤59 years) |  | 0.65 (0.32 – 1.30) | 0.227 |  | 0.58 (0.28 – 1.21) | 0.145 |
|  | Nodal status (pN+ *vs* pN–) |  | 2.29 (1.14 – 4.60) | 0.020 |  | 1.54 (0.74 – 3.21) | 0.246 |
|  | TNM stage (III/IV *vs* I/II) |  | 4.80 (2.09 – 11.0) | 0.000 |  | 5.05 (2.03 – 12.6) | 0.000 |
|  | sHLA-G (Pos. *vs* neg.) |  | 2.76 (0.65 – 11.8) | 0.171 |  | 4.05 (0.89 – 18.4) | 0.071 |
|  |  |  |  |  |  |  |  |
| Adenocarcinoma | Sex (male *vs* female) |  | 1.03 (0.50 – 2.10) | 0.938 |  |  |  |
|  | Age (>61.5 years *vs* ≤61.5years) |  | 0.97 (0.48 – 1.96) | 0.925 |  |  |  |
|  | Nodal status (pN+ *vs* pN–) |  | 2.35 (1.12 – 4.92) | 0.240 |  | 2.01 (0.92 – 4.41) | 0.082 |
|  | TNM stage (III/IV *vs* I/II) |  | 2.56 (1.09 – 5.99) | 0.031 |  | 1.89 (0.76 – 4.67) | 0.170 |
|  | sHLA-G (Pos. *vs* neg.) |  | 1.04 (0.47 – 2.33) | 0.919 |  |  |  |
|  |  |  |  |  |  |  |  |
| Adenosquamous carcinoma | Sex (male *vs* female) |  | 0.67 (0.08 – 5.64) | 0.715 |  |  |  |
|  | Age (>58.5 years *vs* ≤58.5 years) |  | 0.13 (0.02 – 1.10) | 0.061 |  | 0.16 (0.02 – 1.50) | 0.110 |
|  | Nodal status (pN+ *vs* pN–) |  | 3.67 (0.60 – 22.3) | 0.160 |  | 2.51 (0.41 – 15.5) | 0.323 |
|  | TNM stage (III/IV *vs* I/II) |  | 1.04 (0.47 – 2.33) | 0.919 |  |  |  |
|  | sHLA-G (Pos. *vs* neg.) |  | 0.03 (0.00 – 312) | 0.476 |  |  |  |
| Abbreviations: HR=hazard ratio; 95% CI=95% confidence interval; TNM, lymph-node-metastasis and stage according to the TNM classification for esophageal cancer (UICC). *****Cox proportional hazard analysis was used. | | | | | | | |
